# Effect of Carbon Layer Thickness on the Electrocatalytic Oxidation of Glucose in a Ni/BDD Composite Electrode

**DOI:** 10.3390/molecules28155798

**Published:** 2023-08-01

**Authors:** Hangyu Long, Kui Wen, Cuiyin Liu, Xuezhang Liu, Huawen Hu

**Affiliations:** 1 School of Materials Science and Hydrogen Energy, Foshan University, Foshan 528000, China; longhy_csu45@163.com (H.L.); liu_cuiyin@163.com (C.L.); 2 National Engineering Laboratory for Modern Materials Surface Engineering Technology, Guangdong Institute of New Materials, Guangzhou 510651, China; wenkui@gdinm.com; 3 School of Materials and Mechanical Engineering, Jiangxi Science and Technology Normal University, Nanchang 330013, China

**Keywords:** boron-doped diamond, chemical vapor deposition, carbon thickness, glucose, electrochemistry

## Abstract

High-performance non-enzymatic glucose sensor composite electrodes were prepared by loading Ni onto a boron-doped diamond (BDD) film surface through a thermal catalytic etching method. A carbon precipitate with a desired thickness could be formed on the Ni/BDD composite electrode surface by tuning the processing conditions. A systematic study regarding the influence of the precipitated carbon layer thickness on the electrocatalytic oxidation of glucose was conducted. While an oxygen plasma was used to etch the precipitated carbon, Ni/BDD-based composite electrodes with the precipitated carbon layers of different thicknesses could be obtained by controlling the oxygen plasma power. These Ni/BDD electrodes were characterized by SEM microscopies, Raman and XPS spectroscopies, and electrochemical tests. The results showed that the carbon layer thickness exerted a significant impact on the resulting electrocatalytic performance. The electrode etched under 200 W power exhibited the best performance, followed by the untreated electrode and the electrode etched under 400 W power with the worst performance. Specifically, the electrode etched under 200 W was demonstrated to possess the highest sensitivity of 1443.75 μA cm^−2^ mM^−1^ and the lowest detection limit of 0.5 μM.

## 1. Introduction

Blood glucose concentration is an important indicator of many diseases, such as diabetes and endocrine disorders. The blood glucose levels of patients need to be strictly and continuously controlled in a real-time manner. Therefore, the accurate, rapid, and sensitive monitoring of glucose concentration is immensely significant for the diagnosis and treatment of related diseases [1,2]. Enzyme glucose sensors based on glucose oxidase (GOx) are often used to detect glucose levels. However, enzyme sensors exhibit certain drawbacks, including low repeatability, unsatisfactory instability, high cost, and the need for complex manufacturing techniques. Furthermore, the effectiveness of enzyme sensors is influenced by external variables such as humidity, pH, and temperature fluctuations. These limitations restrict the application of enzyme-based sensors [3,4]. Alternatively, non-enzymatic glucose sensors offer excellent characteristics such as cost-effectiveness, high efficiency, high sensitivity, and ease of operation [5]. Particularly, non-enzymatic glucose sensors based on electrochemical methods show remarkable prospects for practical applications [6,7]. Certainly, for non-enzymatic sensors, their sensing performance is largely dictated by electrode materials [8].

As a common non-precious metal for electrode material fabrication, nickel generates the Ni(OH)_2_/NiO(OH) redox pair under alkaline conditions, thus enabling the direct electrocatalytic oxidation of glucose [9]. Compared to other metal nanomaterials (e.g., Pt, Au, Cu, Ag, and CuO), Ni possesses many advantages, such as lower cost, higher resistance to chloride ion poisoning, superior corrosion resistance, and faster electron transfer properties, leading to greater performance and universality in the electrocatalytic oxidation of glucose [10,11]. To further enhance the performance of Ni-based glucose sensors, most researchers have focused on various nanostructures with high specific surface area and surface energy, such as nanoparticles [12,13,14,15], nanowires [16], nanosheets [17], nanowire arrays [18], and three-dimensional nanostructures [10,19,20]. These highly active nanomaterials often need to be loaded onto suitable carrier materials to achieve good dispersion, stability, and rapid electron transfer. Therefore, it is essential to select appropriate carrier materials and loading methods for active nanostructure deposition.

Boron-doped diamond (BDD) is highly promising as an electrode material with a high oxygen evolution potential, high corrosion resistance, and good anti-pollution properties [21]. In the form of solid films for carrying sensitive nanostructures (e.g., Au, Pt, Ag, Cu, or Ni), BDD is structurally stable and can be prepared by a simpler preparation process with better reproducibility compared with other carbon materials such as carbon nanotubes and graphene [22,23]. More importantly, BDD can be directly grown onto substrate electrodes, without involving secondary loading. On the other hand, electrodeposition was mainly adopted to load metal nanoparticles onto BDD. However, the interfacial adhesion between the deposited active nanoparticles and the BDD substrate was weak. During long-term electrochemical testing, weak interfacial binding is incapable to suppress the migration, aggregation, and detachment of certain nanoparticles on the carrier, resulting in unsatisfactory electrode performance [24,25]. Such weak interfacial adhesion also increases the energy barrier for the adsorption and diffusion of analyte molecules at the electrode/solution interface, as well as for electron migration at the interface [26]. To strengthen the interfacial interactions, Dai et al. used nanodiamond-enhanced nucleation to electrodeposit Ni nanoparticles on the BDD surface, which improved the Ni nanoparticle nucleation rate and the binding force between Ni nanoparticles and BDD film [27]. Nevertheless, there is still room remaining to improve the physisorption-based binding between Ni and BDD further. Researchers have attempted to construct chemical binding at the interfaces to enhance the performance of composite electrodes. Hutchison et al. achieved this by modifying the surfaces of Au nanoparticles and BDD electrodes with organic ligands to chemically graft the nanoparticles onto the BDD electrode surface [28]. This approach reduced the energy barrier for electron migration and improved electrode response and stability. However, this method is relatively complex and susceptible to environmental influences during usage.

By employing thermal catalytic etching, Ni can be loaded onto the BDD surface with strong interfacial adhesion. Under high-temperature conditions, Ni is capable of catalytically etching the BDD film, leading Ni particles to embed into BDD and forming a stable interfacial connection between Ni and BDD [29,30]. This approach can resolve the problem of weak interfacial binding associated with electrodeposition and enables the preparation of high-performance non-enzymatic glucose sensor electrodes. Meanwhile, the thermal catalytic etching process results in the precipitation of a substantial amount of carbon, which encapsulates the nanoscale Ni. Research indicates that the precipitated carbon layer can effectively enhance electrode conductivity. In addition, the metal particles can be protected from electrolyte corrosion. A synergistic effect can also be created between the metal and carbon materials, lowering the reaction barrier near the Ni/carbon interface and hence accelerating the oxidation reaction [14,31,32]. Furthermore, some studies suggest that the carbon layer thickness plays a crucial role in regulating the composite electrode performance [33]. However, it is a grand challenge to precisely control the carbon layer thickness from an experimental perspective. Bao et al. reported on the influence of carbon layer thicknesses on carbon nanotube-encapsulated iron-based nanoparticle catalysts for the oxygen reduction reaction [34,35]. The experimental findings revealed that oxygen adhered more strongly to the carbon surface with a smaller thickness, significantly elevating the oxygen reduction reaction rate. While the carbon layer thickness affected the electron transfer rate of the materials, the optimal catalysts typically had a carbon layer with a thickness of only 1–2 layers. The DFT calculations further indicated that an increase in the carbon layer thickness gradually diminished the effect of charge transfer from the metal core to the outermost carbon layer, which became extremely weak beyond three layers. Although the Ni/BDD composite electrode used as a sensor electrode exhibits excellent glucose catalytic performance, the impact of carbon layer thickness on the performance of glucose electrocatalytic oxidation has been unknown until now.

Therefore, in this study, we employed a low-temperature oxygen plasma etching technique to gradually remove the precipitated carbon on the surface of Ni/BDD electrodes, which was generated during the thermal catalytic process. Through a series of material composition characterizations and electrochemical measurements, we systematically analyzed the impact of carbon layer thickness on the electrocatalytic oxidation of glucose. This study presented here sheds light on clarifying the relationship between the precipitated carbon layer thickness and the electrochemical performance of Ni/BDD electrodes and constructing durable and high-performance composite electrode materials with strong interfacial adhesion for biological sensing.

## 2. Results and Discussion

**Figure 1:**
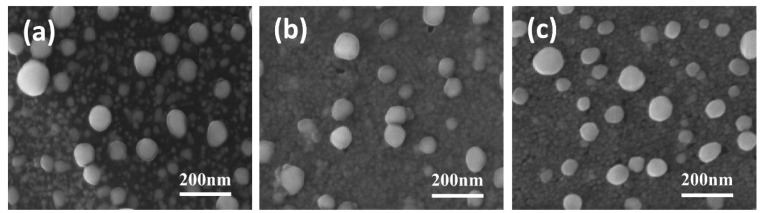
SEM images of (**a**) **untreated**, (**b**) **200 W**, and (**c**) **400 W** electrodes.

Figure 1 shows the SEM images of different Ni/BDD samples. From Figure 1a, it can be observed that, after thermal treatments, large Ni nanoparticles with diameters of approximately 70–100 nm can be observed on the sample surface, in addition to small Ni nanoparticles with diameters of about 10–20 nm that are separated by a dark material and dispersed around the large particles. Upon closer inspection, a translucent coating can be seen on the large particle surface. After 200 W oxygen plasma etching, the translucent coating thickness is reduced (Figure 1b), along with the vanishment of the dark material between particles, enabling the underlying Ni nanoparticles to be exposed and connected. The oxygen plasma etching at 400 W eliminates the translucent coating, making the boundaries between Ni nanoparticles at the bottom clearer (Figure 1c). Based on the thermal catalytic reaction between Ni and diamond, it can be inferred that the dark material and translucent coating are primarily comprised of graphitic phases formed during the catalytic reaction. With an increase in oxygen plasma etching power, the surface graphite layer is partially removed to elevated extents.

The formation of these small Ni nanoparticles at the bottom, together with some large particles on the top surface, reveals that the Ni film exhibits obvious thermal instability with a much lower melting point than bulk Ni with a melting point of 1455 ℃. Compared to conventional bulk materials, nanoscale thin films produced by sputtering have higher surface energy and a larger number of surface atoms. In a low coordination state, these surface atoms possess high reactivity. The much larger surface-to-volume ratio of the nanoparticles with more reactive surface atoms compared with that of bulk materials makes them require much less internal energy to melt [36]. Moreover, the sputtered Ni films contain a high density of grain boundaries, which provide a significant driving force for coarsening and hence result in a lower melting point [37]. This thermal instability significantly increases as the particle size decreases, exhibiting a pronounced size effect. Specifically, coarsening occurs in some nanograined metals (such as Cu) even at ambient temperatures [38]. Such size-related thermal instability, along with the high-temperature impacts and the inherent size effect, causes localized melting on the surface when the heat treatment temperature reaches 700 °C. As a result, diffusion and nucleation of Ni atoms are promoted to aggregate into large particles. On the other hand, the thermal instability accelerates the etching of BDD by Ni at 700 °C and brings rapid precipitation of large amounts of carbon, thus allowing the bottom small Ni particles to be anchored by embedding into the BDD thin film. The rapidly precipitated carbon during the thermal catalytic process immediately encapsulates the nanoscale particles, forming a physical barrier to reduce the aggregation of the bottom Ni nanoparticles and hence resulting in highly dispersed Ni/C nanoclusters.

**Figure 2:**
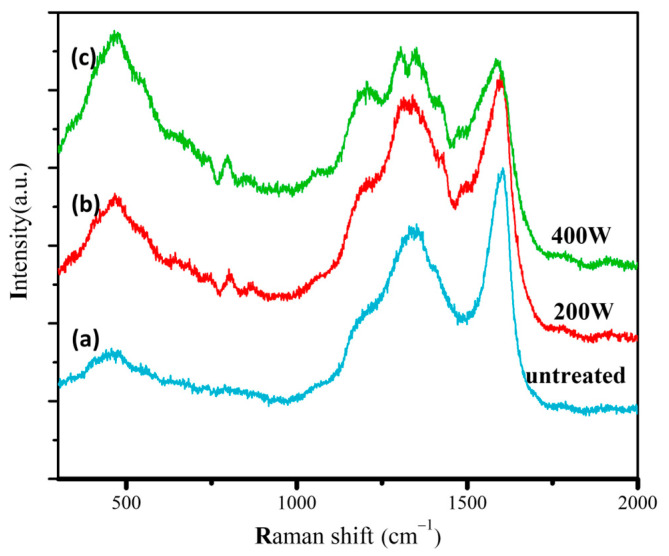
Raman spectra of the (**a**) **untreated**, (**b**) **200 W**, and (**c**) **400 W** electrodes.

Figure 2 shows the Raman spectra of different samples. The characteristic peaks at 500 and 1200 cm^−1^ can be well observed. These two peaks can be attributed to the boron doping in diamonds, which induces a certain degree of lattice disorder, thus confirming the incorporation of boron atoms into the diamond crystal [39,40]. Two distinctive peaks appear at 1350 and 1580 cm^−1^, corresponding to the D peak associated with defects and disordered structures in sp^2^ carbon and the G peak related to the abundance of sp^2^ carbon, respectively. This finding indicates the significant generation of graphite phases after thermal catalytic treatments [41]. With an increase in oxygen plasma etching power from 200 to 400 W, the Raman peaks at 500 and 1200 cm^−1^ gradually intensify, while the characteristic diamond peak at 1332 cm^−1^ becomes more prominent. In contrast, the intensities of the peaks at 1350 and 1580 cm^−1^, which can be indexed to sp^2^ graphite phases, gradually decrease. These results suggest that oxygen plasma etching reduces surface graphite phases, particularly at higher etching power, which reduces the surface graphite phases to a greater extent.

**Figure 3:**
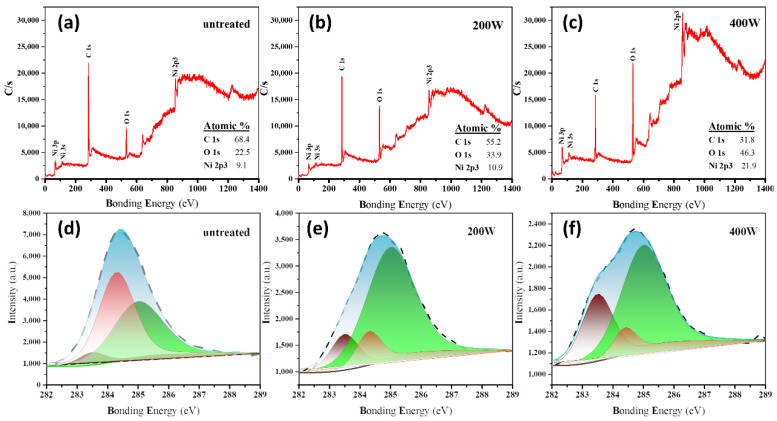
(**a**–**c**) Survey scan and (**d**–**f**) C 1s core-level XPS spectra of (**a**,**d**) **untreated**, (**b**,**e**) **200 W**, and (**c**,**f**) **400 W** samples. The content ratios of C, O, and Ni are calculated based on the C1s, O1s, and Ni 2p3 characteristic peak signals.

In the XPS survey spectra of three different samples, the characteristic peaks at approximately 865, 285, and 532 eV can be assigned to Ni 2p3, C 1s, and O 1s, respectively, thus indicating the presence of Ni, C, and O elements in all samples (Figure 3a–c). In addition, it can be observed that the oxygen plasma etching at a higher power (corresponding to the 400 W sample) enables the characteristic peak signal of the C element to be noticeably decreased whilst strengthening the signals from Ni and O elements. Because XPS is sensitive to surface elements, the etching of carbon atoms from the sample surface by the oxygen plasma weakens the shielding effect on the absorption of underlying Ni. This further indicates that increasing the etching power enhances the exfoliation degree of the carbon layer on the surface.

Figure 3d–f show the XPS spectra and corresponding peak fitting curves of the C 1s for different samples. Three characteristic peaks can be deconvoluted from the high-resolution C 1s XPS spectra. The C1, C2, and C3 peaks at approximately 283.5, 284.4, and 286.6 eV can be attributed to the carbon atom features in carbides [42,43], the C=C bond in sp^2^ hybridized orbitals [44], and the carbon atom features in oxygen-containing functional groups [45], respectively. The oxygen plasma etching steadily enhances the intensity of the C1 peak and the C3 peak while stepwise decreasing the intensity of the C2 peak indexed to the sp^2^ carbon phase with a gradual increase in the etching power. These observations indicate that a significant amount of carbon is oxidized by the plasma and etched from the surface, thus weakening the collected sp^2^ carbon signals in XPS. Additionally, the removal of surface sp^2^ carbon allows the underlying Ni to be exposed, making the collection of Ni signals more efficient, especially in the case of a high oxygen plasma etching power.

**Figure 4:**
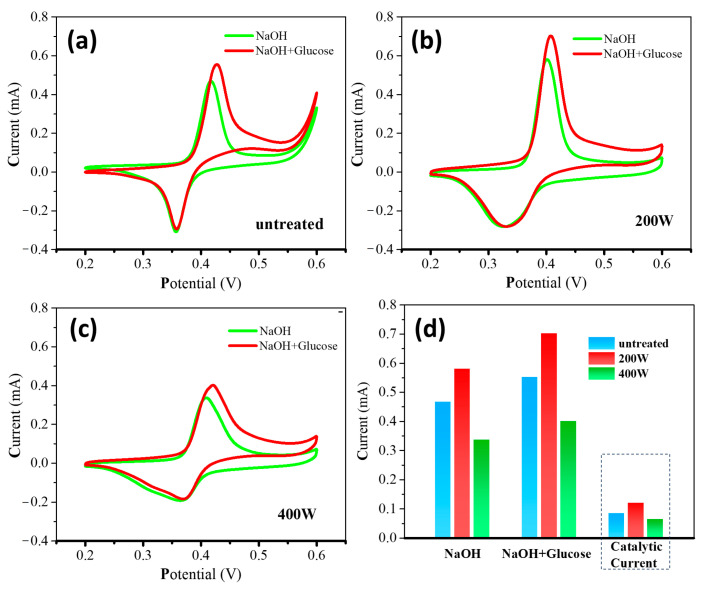
CVs of (**a**) **untreated**, (**b**) **200 W**, and (**c**) **400 W** electrodes in the absence (green line) and presence (red line) of 1 mM glucose in 0.5 M NaOH at 50 mVs^−1^. (**d**) The corresponding oxidation current and catalytic current of different electrodes.

Figure 4 shows the cyclic voltammograms (CV) of different electrodes in a 0.5 M NaOH electrolyte solution and a mixed solution containing 1 mM glucose and a 0.5 M NaOH electrolyte. Before conducting CV tests, a CV pretreatment in a 0.5 M NaOH solution had been carried out. In the CV curves, a pair of Ni(OH)_2_/NiO(OH) redox peaks can be observed. Upon the addition of glucose, the oxidation and reduction peak currents increase and decrease, respectively, along with a positive shift of the oxidation peak potential. This behavior can be attributed to the reduction of some NiOOH species by glucose molecules during the reaction, resulting in a decrease of NiO(OH) and an increase of Ni(OH)_2_. The reaction equations can be expressed as follows [46]:

Ni(OH)_2_ + (OH)^−^ → NiO(OH) + H_2_O + e^−^ (1)

Ni(OH) + glucose → Ni(OH)_2_ + gluconolactone (2)

By calculating the difference in oxidation peak currents in the 0.5 M NaOH solution and the mixture of the 0.5 M NaOH and 1 mM glucose solution, the electrocatalytic currents were obtained, as shown in Figure 4d. A comparison study of the oxidation and electrocatalytic currents presents that the **200 W** electrode exhibits the highest currents, followed by the **untreated** electrode and the **400 W** electrode with the lowest currents. Therefore, the order of the electrocatalytic performance of the three electrodes towards glucose oxidation can be determined as follows: **200 W** > **untreated** > **400 W**.

**Figure 5:**
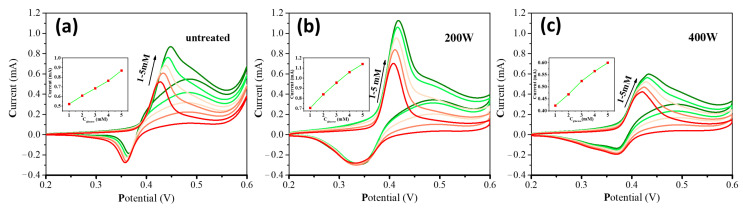
CVs of different electrodes (**a**) **untreated**, (**b**) **200 W**, and (**c**) **400 W** in a 0.5 M NaOH electrolyte with different concentrations of glucose (i.e., 1, 2, 3, 4, and 5 mM) at 50 mVs^−1^. Insets are the corresponding linear fitting curve of oxidation currents vs. glucose concentrations.

Figure 5 shows the CV curves of different electrodes in a mixture solution of 0.5 M NaOH and glucose at varying concentrations (i.e., 1, 2, 3, 4, and 5 mM). With the increase of glucose concentrations, the oxidation peak current steadily increases, and the oxidation peak potential shifts towards more positive values. This result can be attributed to the diffusion limitation of glucose molecules on the electrode surface [47]. The gradual decrease of the reduction peak current can be due to the reduction of the NiOOH species by glucose molecules [48]. The plots of oxidation peak currents as a function of glucose concentrations can be well linearly fitted for all the cases with different electrodes.

**Figure 6:**
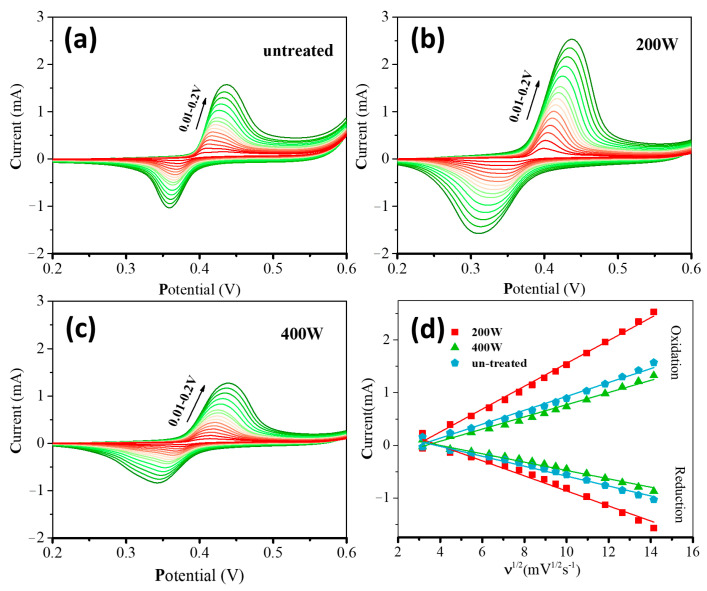
CVs of different electrodes in 0.5 M NaOH with 1 mM glucose at different scan rates (from inner to outer): 10, 20, 30, 40, 50, 60, 70, 80, 90, 100, 120, 140, 160, 180, and 200 mVs^−1^; (**a**) **untreated**, (**b**) **200 W**, and (**c**) **400 W**. (**d**) The linear calibration curves of peak currents vs. the square root of scan rate.

Figure 6a–c depicts the CV curves of different electrodes in a mixture solution of 0.5 M NaOH and 1 mM glucose at various scan rates. As the scan rate increases, the oxidation peak potential continuously shifts towards more positive values. This phenomenon can be attributed to the fact that the electrode requires a larger overpotential to achieve the same electron transfer rate at higher scan rates. Figure 6d presents the fitting curves of the oxidation and reduction peak currents vs. the square root of the scan rate for the systems equipped with different electrodes. The oxidation-reduction currents can be estimated in the order: **200 W** > **untreated** > **400 W**. The peak currents are proportional to the square root of the scan rate for all the systems with different electrodes. This observation indicates that the rate-determining step during the oxidation-reduction process of glucose lies in the diffusion mass transfer [49].

**Figure 7:**
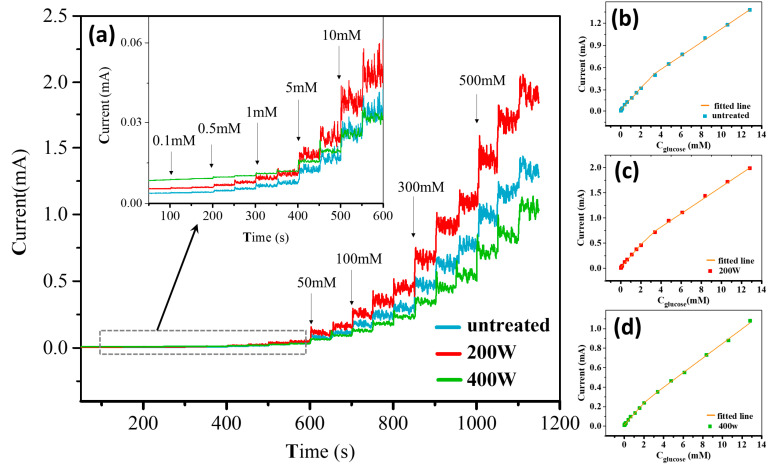
(**a**) Amperometric responses of different electrodes after successive additions of glucose with different concentrations in a 0.5 M NaOH electrolyte. The applied potential is 0.5 V and the dropwise volume is constant at 1 mL. The inset in (**a**) is enlarged curves for the cases with different electrodes. (**b**–**d**) Linear fitting curves of current responses vs. glucose concentrations for different systems with the (**b**) **untreated**, (**c**) **200 W**, and (**d**) **400 W** electrodes.

Figure 7 shows the amperometric responses of different electrodes in a 0.5 M NaOH electrolyte after successively adding glucose with different concentrations. The initial volume of the blank solution was 200 mL, and the solution was continuously stirred during the test. The current response of these electrodes can be found in the following order: **200 W** > **untreated** > **400 W**. The obtained results that were based on the amperometric method to detect glucose are consistent with the electrocatalytic current response observed from the CV curves.

To obtain the calibration curve of the current response as a function of glucose concentrations, we linearly fitted the steady-state current responses of all electrodes against glucose concentrations. The fitting curves, shown in Figure 7b–d, can be divided into two linear regions corresponding to the low (0–2 mM) and high concentration (2–12.8 mM) ranges. The linear fitting equations used to evaluate the electrode calibration curves and their corresponding sensing performances are presented in Table 1. It can be observed that the sensitivities follow the order of **200 W** > **untreated** > **400 W** for both the low and high concentration ranges, with the **200 W** electrode exhibiting the highest sensitivity of 1443.75 μA cm^−2^ mM^−1^. The limit of detection (LOD) can be calculated for each electrode according to the formula LOD = 3SD/S, where SD represents the standard deviation of the background current obtained from 11 repeated measurements in the blank solution (0.5 M NaOH) and S represents the sensitivity. The LOD results are also shown in Table 1, with the detection limit found to be in the following order: **400 W** > **untreated** > **200 W**; the **200 W** electrode exhibits the lowest detection limit of 0.5 μM. Additionally, a comparison between the present Ni/BDD electrode and some typical Ni-based electrodes previously reported elsewhere in non-enzymatic electrochemical sensing of glucose is summarized in Table 2. The results show that the Ni/BDD composite electrode prepared in this work exhibits high sensitivity and low LOD over a wide linear range, thus demonstrating the advance in fabricating superior Ni-based electrodes for non-enzymatic electrochemical sensing of glucose.

The electrochemical testing results indicate an order of **200 W** > **untreated** > **400 W** in terms of the electrocatalytic performance of these three electrodes, suggesting a significant influence of the thickness of the carbon layer formed by thermal catalytic etching on the performance. The **untreated** electrode demonstrates superior electrocatalytic performance, which can be mainly attributed to the following factors: (1) During the thermal catalytic process, the lowered melting point of the nanoscale Ni film with reactive surface Ni atoms renders a reaction between Ni and the BDD substrate, making carbon atoms rapidly diffuse toward the Ni particles and eventually solidly dissolve into the lattice of the Ni particles. The carbon atoms in a solution with Ni particles precipitate out in the form of other carbon allotropes, such as graphite [30]. Such a carbon layer formed by thermal catalytic etching acts as a coating that provides a physical barrier among the nanometer-sized particles, thus promoting the high dispersion of Ni/C nanoparticles. The inhibited Ni particle aggregation renders a high specific surface area and high catalytic activity of the electrode during the electrocatalytic process [62,63]. (2) The synergy generated between the precipitated carbon layer and Ni nanoparticles can significantly alter the electron density in the carbon layer at the interface, which enhances the charge transfer of the composite electrode and improves the surface electrochemical activity of Ni/carbon [64,65]. However, etching at 200 W to decrease the surface carbon layer thickness leads to an improvement in the electrocatalytic performance. The results obtained via Raman spectroscopy indicate that the thermally catalyzed samples exhibit a strong D peak, indicating the presence of abundant defects and disordered structures in the precipitated carbon layer. These structures serve as diffusion channels for glucose molecules [66,67,68]. As the precipitated carbon layer is thinned through oxygen plasma etching, the reduced carbon layer increases the diffusion channels for glucose, which enhances the permeability and diffusion capacity of the glucose solution. Simultaneously, more active sites at the Ni/C interface are exposed as a result of the synergistic effect, thereby increasing the probability of contact with active sites at the Ni/C interface and enhancing the electrocatalytic activity [69]. However, further increasing the etching power to 400 W substantially removes the carbon layer from the electrode surface, resulting in the elimination of numerous Ni/C active sites and a decrease in the Ni/C synergistic effect. Consequently, the electrode’s activity decreases. Therefore, the effective control of the carbon layer thickness plays a crucial role in obtaining high-performance glucose sensors.

## 3. Materials and Methods

### 3.1. Reagents

Glucose was purchased from Sigma-Aldrich (St. Louis, MO, USA). Sodium hydroxide, acetone, and ethanol (analytical grade) were purchased from Tianjin Recovery Technology Development Co., Ltd. (Tianjin, China). All reagents were stored under standard conditions as required. The solvent used throughout the experiment was high-purity water (resistivity of 18.2 MΩ cm). The reaction gases (i.e., CH_4_, B_2_H_6_, and H_2_) employed were all high-purity grades (99.99%).

### 3.2. Fabrication of Electrodes

**Figure 8:**
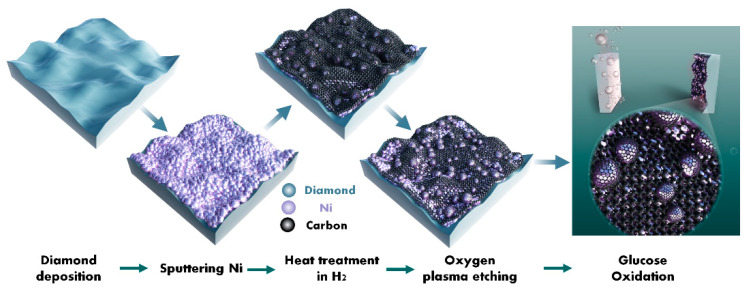
Schematic diagram of the preparation of the Ni/BDD composite electrode through oxygen plasma etching.

Ni/BDD composite electrodes were prepared in a typical process, as shown in Figure 8. Initially, the BDD film was deposited on a P-type heavily doped silicon substrate (4 × 4 × 0.5 mm^3^) by the hot filament chemical vapor deposition technique. Before the deposition, the silicon substrates were subjected to stepwise ultrasonic pretreatments in acetone, a nanodiamond suspension to seed, and ethanol for 10, 30, and 5 min, respectively. The BDD film was then deposited in H_2_ (49 sccm), CH_4_ (1 sccm), and B_2_H_6_ (0.2 sccm) at a temperature of 750 °C and a pressure of 3 kPa for 8 h. Secondly, a nano-thick (about 20 nm) Ni film was deposited onto the formed BDD film by DC magnetron sputtering with a power of 150W in Ar (30 sccm) at a pressure of 0.5 Pa for 20 s. Thirdly, the deposited Ni film sample underwent thermal catalytic treatments in a tubular annealing furnace in H_2_ (100 sccm) at a pressure of 10 kPa and a temperature of 700 ℃ for 30 min. Fourthly, the sample obtained after the thermal catalytic treatment was subjected to oxygen plasma etching at two powers (i.e., 200 and 400 W) for a duration of 5 min. Finally, the electrodes were encapsulated using insulating adhesives, conductive silver pastes, and copper wires for electrochemical testing purposes. The electrode without involving the oxygen plasma treatment was designated as the **untreated** electrode, while the counterparts treated under 200 and 400 W oxygen plasma powers were labeled as **200 W** and **400 W** electrodes, respectively. 

### 3.3. Characterizations

The sample surface morphologies were observed by scanning electron microscopy (SEM) (FEI, Hillsboro, OR, USA, Nova NanoSEM 230). The compositions of the prepared electrodes were characterized by Raman spectroscopy (HORIBA, Paris, France, LabRAM HR800; 532 nm, 10 mW) and X-ray photoelectron spectroscopy (XPS, Thermo Fisher-VG Scientific, Waltham, MA, USA, ESCALAB250Xi).

### 3.4. Electrochemical Performance Analysis

Electrochemical testing was performed using a CHI660E electrochemical workstation at room temperature (25 ℃). A standard three-electrode system was adopted, with an Ag/AgCl, a platinum sheet (10 × 10 × 1 mm^3^), and the prepared electrode as the reference, counter, and working electrodes, respectively. Before each measurement, all electrodes underwent cyclic voltammograms (CV) in a 0.5 M NaOH solution within the potential range of 0.2 to 0.6 V for 100 cycles to achieve a stable Ni(OH)_2_/NiOOH layer. After the pretreatment, all electrodes were immersed in ultrapure water with a resistivity of 18.2 MΩ.

## 4. Conclusions

In this study, the influence of the carbon layer thickness of Ni/BDD composite electrodes on the electrocatalytic oxidation of glucose was analyzed systematically. Ni/BDD composite electrodes obtained through thermal catalytic etching were subjected to oxygen plasma etching to control the surface carbon layer thickness by varying the etching power from 200 to 400 W. The increase in the etching power resulted in the reduction of the thickness of the surface precipitated carbon. The **200 W** electrode, subjected to a milder etching, exhibited the best electrochemical performance, followed by the **untreated** electrode with the thickest carbon layer, while the **400 W** electrode with the thinnest carbon layer showed the worst electrochemical performance. Two linear dependencies of current responses vs. glucose concentrations were detected for the **200 W** electrode in the glucose concentration range of 0–2 mM and 2–12.8 mM, with the sensitivity measured as 1443.75 and 831.25 μA mM^−1^ cm^−2^, respectively. The **200 W** electrode also exhibited the lowest LOD of 0.5 μM (S/N = 3). All of the demonstrations indicated that the carbon layer thickness exerted a significant impact on the electrocatalytic performance in glucose sensing. This work will establish a solid foundation for mediating the precipitated carbon layer thickness to optimize the structure and properties of BDD-based composite electrodes for a wide range of applications even beyond biological sensing.

## Figures and Tables

**Table 1:** Comparison of the glucose sensing performance of different electrodes prepared in this study.

Electrodes	Linear Range (mM)	Sensitivity (μA mM^−1^ cm^−2^)	LOD (μM)	Linear Calibration Equation
untreated	0~2	1012.5	1.2	I(mA) = 0.162C + 0.0084
	2~12.8	568.75		I(mA) = 0.091C + 0.214
200 W	0~2	1443.75	0.5	I(mA) = 0.231C + 0.0125
	2~12.8	831.25		I(mA) = 0.133C + 0.287
400 W	0~2	706.25	1.6	I(mA) = 0.113C + 0.0123
	2~12.8	468.75		I(mA) = 0.075C + 0.0098

**Table 2:** Comparison of the present Ni/BDD electrode with the previously reported Ni-based electrodes in non-enzymatic electrochemical sensing of glucose, including linear range, sensitivity, and LOD.

Electrode Materials	Linear Range (mM)	Sensitivity (μA mM^−1^ cm^−2^)	LOD (μM)	Ref.
Ni-NPs ^a^/NCNs ^b^-500	0.0001–0.5336	337.32	0.07	[50]
	0.5336–3.03	210.56		
Ni-NPs/NC ^c^	0.002–4.658	660.3	0.12	[51]
Ni-NPs/MOF ^d^	0.004–5.664	367.45	0.8	[52]
Ni/Ni foam	0.01–0.7	2370	5	[53]
Ni(II)-CP ^e^/C60	0.01–3	614.29	4.3	[54]
	3–11			
Ni30/PF ^f^	0.02–0.5	670	8	[55]
Ni@C@rGO ^g^	0.002–0.951	1211.41	0.34	[56]
Ni@C/3D-KSCs ^h^	0.024–1.2	9.11	7.85	[57]
NiO/Ni foil	0.0005–9	4400	0.007	[58]
Ni(OH)_2_/IN625 foam ^i^	0.001–10	5685	2	[59]
Ni(OH)_2_/Ni foam	0.002–0.04	1130	1	[60]
Ni-ND ^j^/BDD	0.0002–0.012	120	0.05	[27]
	0.0313–1.06	35.6		
Ni-Microparticles/BDD	0.1–10	1040	2.7	[25]
Au-Ni/BDD	0.02–2	157.5	0.0026	[61]
	2–9	61.2		
Ni/BDD	0–2	1443.75	0.5	This work
	2–12.8	831.25		

^a^ NPs: nanoparticles; ^b^ MCNs: nanoporous carbon nanorods; ^c^ NC: nitrogen-doped carbon; ^d^ MOF: metal–organic framework; ^e^ CP: coordination polymer; ^f^ PF: pyrogallol-formaldehyde; ^g^ rGO: reduced graphene oxide; ^h^ 3D-KSCs: 3D kenaf stem-derived porous carbon; ^i^ IN625 foam: a three-dimensional Inconel 625 foam; ^j^ ND: nanodiamond.

## Data Availability

Data are available with requirements.

## References

[1] Zhao Z., Huang Y., Li Q., Mei H., Zhu F., Gong W. (2021). Electrochemical glucose sensitive device based on graphene supported Co_3_O_4_@Ag NWs core-shell nanostructures. Appl. Surf. Sci..

[2] Baek S.H., Roh J., Park C.Y., Kim M.W., Shi R., Kailasa S.K., Park T.J. (2020). Cu-nanoflower decorated gold nanoparticles-graphene oxide nanofiber as electrochemical biosensor for glucose detection. Mater. Sci. Eng. C-Mater..

[3] Deshmukh M.A., Kang B.C., Ha T.J. (2020). Non-enzymatic electrochemical glucose sensors based on polyaniline/reduced-graphene-oxide nanocomposites functionalized with silver nanoparticles. J. Mater. Chem. C.

[4] Romeo A., Moya A., Leung T.S., Gabriel G., Villa R., Sánchez S. (2018). Inkjet printed flexible non-enzymatic glucose sensor for tear fluid analysis. Appl. Mater. Today.

[5] Hu H., Zavabeti A., Quan H., Zhu W., Wei H., Chen D., Ou J.Z. (2019). Recent advances in two-dimensional transition metal dichalcogenides for biological sensing. Biosens. Bioelectron..

[6] Lai T., Peng S., Shu H., Chen T., Xiao X., Wang Y. (2022). A High-Performance Non-Enzymatic Sensor Based on Nickel Foam Decorated with Co-CdIn_2_O_4_ Nanoparticles for Electrochemical Detection of Glucose and Its Application in Human Serum. J. Electrochem. Soc..

[7] Bairagi P.K., Verma N. (2019). Electro-polymerized polyacrylamide nano film grown on a Ni-reduced graphene oxide- polymer composite: A highly selective non-enzymatic electrochemical recognition element for glucose. Sens. Actuators B-Chem..

[8] Farid A., Zhonghua C., Khan A.S., Javid M., Khan I.A., Khan A.A., Fan Z., Pan L. (2023). Ni_3_V_2_O_8_ Nanosheets Grafted on 3D Helical-shaped Carbon Nanocoils as A Binder-free Hierarchical Composite for Efficient Non-enzymatic Glucose Sensing. Adv. Funct. Mater..

[9] Miao Y., Ouyang L., Zhou S., Xu L., Yang Z., Xiao M., Ouyang R. (2014). Electrocatalysis and electroanalysis of nickel, its oxides, hydroxides and oxyhydroxides toward small molecules. Biosens. Bioelectron..

[10] Niu X., Lan M., Zhao H., Chen C. (2013). Highly Sensitive and Selective Nonenzymatic Detection of Glucose Using Three-Dimensional Porous Nickel Nanostructures. Anal. Chem..

[11] Weremfo A., Fong S.T.C., Khan A., Hibbert D.B., Zhao C. (2017). Electrochemically roughened nanoporous platinum electrodes for non-enzymatic glucose sensors. Electrochim. Acta.

[12] Lin K.C., Lin Y.C., Chen S.M. (2013). A highly sensitive nonenzymatic glucose sensor based on multi-walled carbon nanotubes decorated with nickel and copper nanoparticles. Electrochim. Acta.

[13] Gong Z., Hu N., Ye W., Zheng K., Li C., Ma L., Wei Q., Yu Z., Zhou K., Huang N. (2019). High-performance non-enzymatic glucose sensor based on Ni/Cu/boron-doped diamond electrode. J. Electroanal. Chem..

[14] Zeng S., Wei Q., Long H., Meng L., Pei E.S. (2020). Annealing temperature regulating the dispersity and composition of nickel-carbon nanoparticles for enhanced glucose sensing. J. Electroanal. Chem..

[15] Jothi L., Jayakumar N., Jaganathan S.K., Nageswaran G. (2018). Ultrasensitive and selective non-enzymatic electrochemical glucose sensor based on hybrid material of graphene nanosheets/graphene nanoribbons/nickel nanoparticle. Mater. Res. Bull..

[16] Qin L., He L., Zhao J., Zhao B., Yin Y., Yang Y. (2017). Synthesis of Ni/Au multilayer nanowire arrays for ultrasensitive non-enzymatic sensing of glucose. Sens. Actuators B-Chem..

[17] Zhang L., Ding Y., Li R., Ye C., Zhao G., Wang Y. (2017). Ni-Based metal-organic framework derived Ni@C nanosheets on a Ni foam substrate as a supersensitive non-enzymatic glucose sensor. J. Mater. Chem. B.

[18] Watanabe T., Einaga Y. (2009). Design and fabrication of nickel microdisk-arrayed diamond electrodes for a non-enzymatic glucose sensor based on control of diffusion profiles. Biosens. Bioelectron..

[19] Vennila P., Yoo D.J., Kim A.R. (2017). Ni-Co/Fe_3_O_4_ flower-like nanocomposite for the highly sensitive and selective enzyme free glucose sensor applications. J. Alloys Compd..

[20] Xu H., Xia C., Wang S., Han F., Akbari M.K., Hai Z., Zhuiykov S. (2018). Electrochemical non-enzymatic glucose sensor based on hierarchical 3D Co_3_O_4_/Ni heterostructure electrode for pushing sensitivity boundary to a new limit. Sens. Actuators B-Chem..

[21] Muzyka K., Sun J., Fereja T.H., Lan Y., Zhang W., Xu G. (2019). Boron-doped diamond: Current progress and challenges in view of electroanalytical applications. Anal. Methods.

[22] Liang Y., Xu X., Yuan F., Lin Y., Xu Y., Zhang Y., Chen D., Wang W., Hu H., Ou J.Z. (2023). Graphene oxide additive-driven widening of microporous biochar for promoting water pollutant capturing. Carbon.

[23] Hu H., Ou J.Z., Xu X., Lin Y., Zhang Y., Zhao H., Chen D., He M., Huang Y., Deng L. (2021). Graphene-assisted construction of electrocatalysts for carbon dioxide reduction. Chem. Eng. J..

[24] Hutton L.A., Vidotti M., Patel A.N., Newton M.E., Unwin P.R., Macpherson J.V. (2011). Electrodeposition of Nickel Hydroxide Nanoparticles on Boron-Doped Diamond Electrodes for Oxidative Electrocatalysis. J. Phys. Chem. C.

[25] Toghill K.E., Xiao L., Phillips M.A., Compton R.G. (2010). The non-enzymatic determination of glucose using an electrolytically fabricated nickel microparticle modified boron-doped diamond electrode or nickel foil electrode. Sens. Actuators B-Chem..

[26] Gao F., Yang N., Nebel C.E. (2013). Highly stable platinum nanoparticles on diamond. Electrochim. Acta.

[27] Dai W., Li M., Gao S., Li H., Li C., Xu S., Wu X., Yang B. (2016). Fabrication of Nickel/nanodiamond/boron-doped diamond electrode for non-enzymatic glucose biosensor. Electrochim. Acta.

[28] Young S.L., Kellon J.E., Hutchison J.E. (2016). Small Gold Nanoparticles Interfaced to Electrodes through Molecular Linkers: A Platform to Enhance Electron Transfer and Increase Electrochemically Active Surface Area. J. Am. Chem. Soc..

[29] Deng Z., Long H., Wei Q., Yu Z., Zhou B., Wang Y., Zhang L., Li S., Ma L., Xie Y. (2017). High-performance non-enzymatic glucose sensor based on nickel-microcrystalline graphite-boron doped diamond complex electrode. Sens. Actuators B-Chem..

[30] Li S., Ma L., Long H., Yu X., Luo H., Wang Y., Zhu H., Yu Z., Ma M., Wei Q. (2016). Enhanced electron field emission properties of diamond/microcrystalline graphite composite films synthesized by thermal catalytic etching. Appl. Surf. Sci..

[31] Liu Y., Liu J., Wang J., Banis M.N., Xiao B., Lushington A., Xiao W., Li R., Sham T.-K., Liang G. (2018). Formation of size-dependent and conductive phase on lithium iron phosphate during carbon coating. Nat. Commun..

[32] Yao Y., Chen H., Lian C., Wei F., Zhang D., Wu G., Chen B., Wang S. (2016). Fe, Co, Ni nanocrystals encapsulated in nitrogen-doped carbon nanotubes as Fenton-like catalysts for organic pollutant removal. J. Hazard. Mater..

[33] Peng Y., Chen S. (2018). Electrocatalysts Based on Metal@Carbon Core@Shell Nanocomposites: An Overview. Green Energy Environ..

[34] Deng D., Yu L., Chen X., Wang G., Jin L., Pan X., Deng J., Sun G., Bao X. (2013). Iron Encapsulated within Pod-like Carbon Nanotubes for Oxygen Reduction Reaction. Angew. Chem. Int. Ed..

[35] Deng J., Yu L., Deng D., Chen X., Yang F., Bao X. (2013). Highly active reduction of oxygen on a FeCo alloy catalyst encapsulated in pod-like carbon nanotubes with fewer walls. J. Mater. Chem. A.

[36] Delgado-Callico L., Rossi K., Pinto-Miles R., Salzbrenner P., Baletto F. (2021). A universal signature in the melting of metallic nanoparticles. Nanoscale.

[37] Zhou X., Li X.Y., Lu K. (2018). Enhanced thermal stability of nanograined metals below a critical grain size. Science.

[38] Huang Y., Sabbaghianrad S., Almazrouee A.I., Al-Fadhalah K.J., Alhajeri S.N., Langdon T.G. (2016). The significance of self-annealing at room temperature in high purity copper processed by high-pressure torsion. Mater. Sci. Eng. A.

[39] Long H., Luo H., Luo J., Xie Y., Deng Z., Zhang X., Wang Y., Wei Q.P., Yu Z.M. (2015). The concentration gradient of boron along the growth direction in boron doped chemical vapor deposited diamond. Mater. Lett..

[40] Mortet V., Taylor A., Vlčková Živcová Z., Machon D., Frank O., Hubík P., Tremouilles D., Kavan L. (2018). Analysis of heavily boron-doped diamond Raman spectrum. Diam. Relat. Mater..

[41] Knight D.S., White W.B. (1989). Characterization of diamond films by Raman spectroscopy. J. Mater. Res..

[42] Wiltner A., Linsmeier C. (2004). Formation of endothermic carbides on iron and nickel. Phys. Status Solidi (A).

[43] Qiu H.J., Du P., Hu K., Gao J., Li H., Liu P., Ina T., Ohara K., Ito Y., Chen M. (2019). Metal and Nonmetal Codoped 3D Nanoporous Graphene for Efficient Bifunctional Electrocatalysis and Rechargeable Zn-Air Batteries. Adv. Mater..

[44] Ayres Z.J., Borrill A.J., Newland J.C., Newton M.E., Macpherson J.V. (2016). Controlled sp_2_ Functionalization of Boron Doped Diamond as a Route for the Fabrication of Robust and Nernstian pH Electrodes. Anal. Chem..

[45] Ryl J., Burczyk L., Bogdanowicz R., Sobaszek M., Darowicki K. (2016). Study on surface termination of boron-doped diamond electrodes under anbdic polarization in H_2_SO_4_ by means of dynamic impedance technique. Carbon.

[46] Jiang Y., Yu S., Li J., Jia L., Wang C. (2013). Improvement of sensitive Ni(OH)_2_ nonenzymatic glucose sensor based on carbon nanotube/polyimide membrane. Carbon.

[47] Lu L.M., Zhang L., Qu F.L., Lu H.X., Zhang X.B., Wu Z.S., Huan S.Y., Wang Q.A., Shen G.L., Yu R.Q. (2009). A nano-Ni based ultrasensitive nonenzymatic electrochemical sensor for glucose: Enhancing sensitivity through a nanowire array strategy. Biosens. Bioelectron..

[48] Kleijn S.E.F., Lai S.C.S., Koper M.T.M., Unwin P.R. (2014). Electrochemistry of Nanoparticles. Angew. Chem. Int. Ed..

[49] Karuppiah C., Velmurugan M., Chen S.M., Tsai S.H., Lou B.S., Ajmal Ali M., Al Hemaid F.M.A. (2015). A simple hydrothermal synthesis and fabrication of zinc oxide-copper oxide heterostructure for the sensitive determination of nonenzymatic glucose biosensor. Sens. Actuators B-Chem..

[50] Jia H., Shang N., Feng Y., Ye H., Zhang Y. (2021). Facile preparation of Ni nanoparticle embedded on mesoporous carbon nanorods for non-enzymatic glucose detection. J. Colloid Interface Sci..

[51] Gao W., Li Q., Dou M., Zhang Z., Wang F. (2018). Self-supported Ni nanoparticles embedded on nitrogen-doped carbon derived from nickel polyphthalocyanine for high-performance non-enzymatic glucose detection. J. Mater. Chem. B.

[52] Shu Y., Yan Y., Chen J., Xu Q., Pang H., Hu X. (2017). Ni and NiO Nanoparticles Decorated Metal–Organic Framework Nanosheets: Facile Synthesis and High-Performance Nonenzymatic Glucose Detection in Human Serum. ACS Appl. Mater. Interfaces.

[53] Iwu K.O., Lombardo A., Sanz R., Scirè S., Mirabella S. (2016). Facile synthesis of Ni nanofoam for flexible and low-cost non-enzymatic glucose sensing. Sens. Actuators B-Chem..

[54] Shahhoseini L., Mohammadi R., Ghanbari B., Shahrokhian S. (2019). Ni(II) 1D-coordination polymer/C60-modified glassy carbon electrode as a highly sensitive non-enzymatic glucose electrochemical sensor. Appl. Surf. Sci..

[55] Marini S., Ben Mansour N., Hjiri M., Dhahri R., El Mir L., Espro C., Bonavita A., Galvagno S., Neri G., Leonardi S.G. (2018). Non-enzymatic Glucose Sensor Based on Nickel/Carbon Composite. Electroanalysis.

[56] Hao J., Li C., Wu C., Wu K. (2019). In-situ synthesis of carbon-encapsulated Ni nanoparticles decorated graphene nanosheets with high reactivity toward glucose oxidation and sensing. Carbon.

[57] Wang L., Peng C., Yang H., Miao L., Xu L., Wang L., Song Y. (2019). Ni@carbon nanocomposites/macroporous carbon for glucose sensor. J. Mater. Sci..

[58] Singer N., Pillai R.G., Johnson A.I.D., Harris K.D., Jemere A.B. (2020). Nanostructured nickel oxide electrodes for non-enzymatic electrochemical glucose sensing. Microchim. Acta.

[59] Kihal R., Fisli H., Chelaghmia M.L., Drissi W., Boukharouba C., Abdi S., Nacef M., Affoune A.M., Pontié M. (2023). A novel and ultrasensitive non-enzymatic electrochemical glucose sensor in real human blood samples based on facile one-step electrochemical synthesis of nickel hydroxides nanoparticles onto a three-dimensional Inconel 625 foam. J. Appl. Electrochem..

[60] Zhao Y., Gu G., You S., Ji R., Suo H., Zhao C., Liu F. (2015). Preparation of Ni(OH)_2_ nanosheets on Ni foam via a direct precipitation method for a highly sensitive non-enzymatic glucose sensor. Rsc Adv..

[61] Yao K., Dai B., Tan X., Ralchenko V., Yang L., Liu B., Su Z., Zhao J., Liu K., Han J. (2020). Fabrication of Au/Ni/boron-doped diamond electrodes via hydrogen plasma etching graphite and amorphous boron for efficient non-enzymatic sensing of glucose. J. Electroanal. Chem..

[62] Lee H.K., Sung Y.E., Choi I., Lim T., Kwon O.J. (2017). Novel synthesis of highly durable and active Pt catalyst encapsulated in nitrogen containing carbon for polymer electrolyte membrane fuel cell. J. Power Sources.

[63] Ahn C.Y., Hwang W., Lee H., Kim S., Park J.E., Kim O.H., Her M., Cho Y.H., Sung Y.E. (2018). Effect of N-doped carbon coatings on the durability of highly loaded platinum and alloy catalysts with different carbon supports for polymer electrolyte membrane fuel cells. Int. J. Hydrogen Energy.

[64] Deng J., Ren P., Deng D., Bao X. (2015). Enhanced Electron Penetration through an Ultrathin Graphene Layer for Highly Efficient Catalysis of the Hydrogen Evolution Reaction. Angew. Chem. Int. Ed..

[65] Jang J.H., Jeffery A.A., Min J., Jung N., Yoo S.J. (2021). Emerging carbon shell-encapsulated metal nanocatalysts for fuel cells and water electrolysis. Nanoscale.

[66] Chung D.Y., Jun S.W., Yoon G., Kwon S.G., Shin D.Y., Seo P., Yoo J.M., Shin H., Chung Y.H., Kim H. (2015). Highly Durable and Active PtFe Nanocatalyst for Electrochemical Oxygen Reduction Reaction. J. Am. Chem. Soc..

[67] Choi C.H., Kwon H.C., Yook S., Shin H., Kim H., Choi M. (2014). Hydrogen Peroxide Synthesis via Enhanced Two-Electron Oxygen Reduction Pathway on Carbon-Coated Pt Surface. J. Phys. Chem. C.

[68] Genorio B., Harrison K.L., Connell J.G., Dražić G., Zavadil K.R., Markovic N.M., Strmcnik D. (2019). Tuning the Selectivity and Activity of Electrochemical Interfaces with Defective Graphene Oxide and Reduced Graphene Oxide. ACS Appl. Mater. Interfaces.

[69] Yoo J.M., Shin H., Chung D.Y., Sung Y.E. (2022). Carbon Shell on Active Nanocatalyst for Stable Electrocatalysis. Acc. Chem. Res..

